# Snail mucus from the mantle and foot of two land snails, *Lissachatina fulica* and *Hemiplecta distincta*, exhibits different protein profile and biological activity

**DOI:** 10.1186/s13104-021-05557-0

**Published:** 2021-04-15

**Authors:** Nattaphop Noothuan, Kantamas Apitanyasai, Somsak Panha, Anchalee Tassanakajon

**Affiliations:** 1 Department of Biochemistry, Faculty of Science, Chulalongkorn University, Bangkok, 10330 Thailand; 2 Animal Systematics Research Unit, Department of Biology, Faculty of Science, Chulalongkorn University, Bangkok, 10330 Thailand

**Keywords:** Land snail, Snail mucus, Antimicrobial activity, Anti-tyrosinase activity, Antioxidant activity

## Abstract

**Objective:**

Snails secrete different types of mucus that serve several functions, and are increasingly being exploited for medical and cosmetic applications. In this study, we explored the protein pattern and compared the biological properties of the mucus secreted from the mantle collar and foot of two snail species, *Lissachatina fulica* and *Hemiplecta distincta*.

**Result:**

Protein profile showed a different pattern between the two species and between the two secretory parts. The mantle-specific protein bands were further characterized and among them was an antibacterial protein, achacin. Accordingly, the mucus from the mantle exhibited the higher antibacterial activity than that from the foot in both snail species. The mucus from *H. distincta*, first reported here, also showed antibacterial properties, but with a lower activity compared to that for *L. fulica*. Snail mucus also exhibited anti-tyrosinase activity and antioxidant activity but with no significant difference between the foot and mantle mucus. These results indicate some different protein compositions and biological activities of snail slime from the mantle and foot, which might be associated with their specific functions in the animal and are useful for medical applications.

**Supplementary Information:**

The online version contains supplementary material available at 10.1186/s13104-021-05557-0.

## Introduction

Land snails have been used as food and for various medical treatments for centuries [1–3]. The snail slime (mucus) has many functions in the animal, such as adhesive, emollient, moisturizing, lubricant, and defense [4–7]. Recently, snail slimes have been applied in human medical and cosmetics [8–11]. Studies have shown that snail mucus exhibits various biological activities, such as antimicrobial, antioxidant, anti-tyrosinase, and antitumoral activities [10, 12, 13]. In addition, many compounds have been found in snail mucus, such as allantoin, hyaluronic acid, peptides and proteins [3, 4, 14]. Moreover, it has been reported that different kinds of mucus are released from the different types of secretory glands in a snail, depending on the way it is stimulated [15]. However, little work on the biochemical properties that give rise to these functional differences has been elucidated.

In this study, the mucus was collected from the mantle and foot of two snail species, *H. distincta* and *L fulica*, in which *H. distincta* is distributed in deciduous forest in Thailand [16], but *L. fulica* is a very famous invasive alien species that occurring everywhere at anthropogenic areas in many parts of the world [17]*.* The protein pattern and biological properties (antioxidant, antibacterial, and anti-tyrosinase activities) of the mucus were then compared.

The results revealed somewhat different protein compositions and biological properties that are probably associated with their functions and are useful for cosmetic and medical applications*.*

## Materials and methods

### Sample preparation

Two species of land snail, *L. fulica* and *H. distincta*, were collected from the wild in Thailand. The snails (n = 20 each species/anatomical location) were reared at 24 °C for 2 days then, the mucus was extracted by gently poking on foot and mantle lobes. The harvested mucus was filtered through 0.45-µm membrane (Millipore) and stored at 4 °C before use. This process did not harm the snails and the experiments was carried out in accordance with the approved guidelines of the Animal Care and Use Committee of Faculty of Science, Chulalongkorn University (Protocol Review No. 1223003).

*Escherichia coli* ATCC 25922, *Bacillus subtilis* ATCC 6633, and *Acinetobacter* spp. L9, PK3, and Y3 were cultured in Luria-Bertani broth at 37 °C, while *Staphylococcus aureus* ATCC 25923 was cultured in tryptic soy broth with 2% (w/v) NaCl at 30 °C with shaking overnight. The bacteria were re-inoculated in the fresh media and cultured until they reached OD_600_ of 0.2 and were diluted about hundred-fold in poor broth [1% (w/v) tryptone, 0.5% (w/v) NaCl, pH 7.5] to an OD_600_ of 0.001. The selected bacteria are prevalent species of gram negative and positive bacteria and most common nosocomial pathogens.

### SDS-PAGE and LC-MS/MS

The protein concentration of mucus was determined using the Bradford assay [18], and then 25 µg of the sample was mixed with 5X reducing SDS loading dye and heated at 100 °C for 10 min. The samples were then subjected to 12.5% SDS-PAGE. One gel was stained with Coomassie blue and the other gel was developed by silver staining [19].

The selected protein bands were cut from the stained SDS-PAGE and analyzed using LC-MS/MS at the Research Instrument Center, Khon Kaen University, Thailand. Briefly, the protein bands were destained, reduced, and digested with Sequencing Grade Modified Trypsin. The tryptic peptides were extracted and analyzed using LC-MS/MS. The obtained data were searched in Protein database using the MASCOT program.

### Antimicrobial activity assay

The antimicrobial activity of snail mucus was analyzed using the minimal inhibitory concentration assay [20]. The snail mucus (12.5, 25, 50, 100, and 200 µg of total protein in 100 µl phosphate-buffered saline (PBS)) was added and then inoculated with 20 µl of the fresh bacterial culture (1 × 10^4^ cells, OD_600_ = 0.001). The reactions were incubated overnight and then the bacterial growth was measured at OD_600._ The negative controls were performed using PBS.

### Anti-tyrosinase activity assay

Each 25-µg total protein of mucus adjusted with PBS to 70 µl was incubated with 10 µl of mushroom tyrosinase (Sigma-Aldrich) in potassium phosphate buffer pH 6.5 (5 U/reaction), and then 20 µl of 1 mg/ml L-DOPA (Sigma-Aldrich) in buffer was added. The reactions were monitored the absorbance at *A*_475_. Distilled water was used as a negative control and kojic acid as positive control. The inhibition was calculated from the formula: Inhibition (%) = [1 − (*A*_475_ in sample/*A*_475_ in control)] × 100%.

### Antioxidant activity assay

A 100-µl of the snail mucus in various concentration was added into the each well and incubated with 100 µl of 0.2 mM DPPH in absolute ethanol in dark place for 30 min. Then, the absorbance was measured at 517 nm (A_517_) [21]. Distilled water was used as a negative control and ascorbic acid as a positive control. The DPPH scavenging effect was calculated by the following formula: %Effective = (*A*_Blank_ – *A*_Sample_)/*A*_sample_ × 100. %Effective were plotted against concentration by GraphPad Prism 8. The EC_50_ values were calculated as the concentration to cause half-maximal inhibition of DPPH radical scavenging.

### Statistical analysis

Results were represented as mean with standard deviation in triplicate. Statistical analysis was performed using a one-way analysis of variance (ANOVA) followed by Tukey’s test. Significance was accepted at the *p* < 0.05 level.

## Results

### Snail mucus extraction and protein profiles

The protein pattern of the two snails analyzed by 12.5% SDS-PAGE exhibited quite a different banding pattern despite some commons bands that were observed (Additional file 1: Fig. S1). The *L. fulica* mucus showed major bands at about 13, 37, 70, and > 200 kDa, whereas *H. distincta* showed major bands at approximately 11, 12, 14, 25, and 120 kDa (Additional file 2: Fig. S2).

### Protein pattern of snail mucus from the mantle and foot of *L. fulica* and *H. distincta*

**Fig.1:**
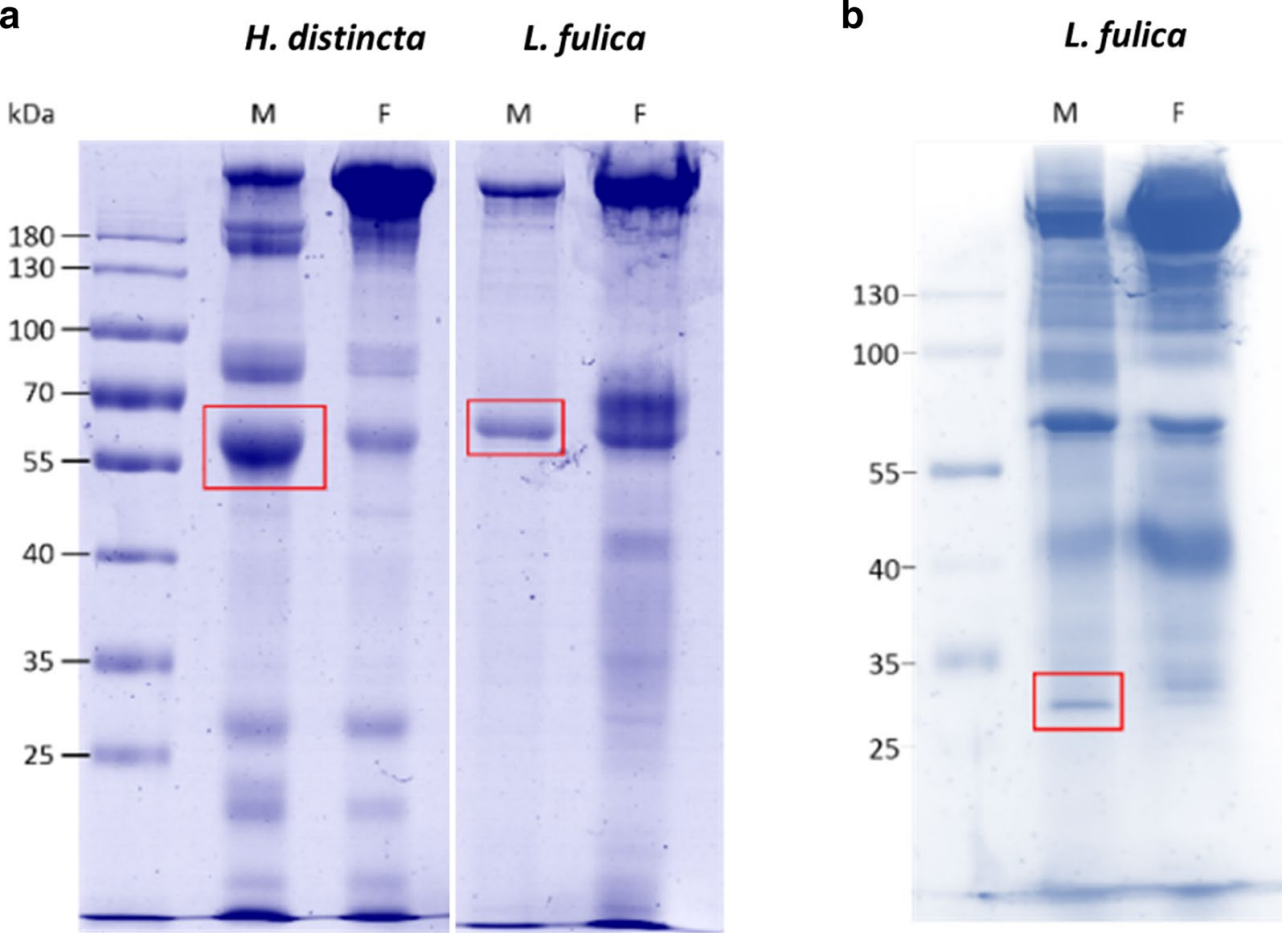
Analysis of the protein pattern in snail mucus from the mantle (M) or foot (F) of *L. fulica* and *H. distincta* by SDS-PAGE. A total protein content of 25 µg/lane was subjected to 12.5% SDS-PAGE and stained with **a** Coomassie Brilliant Blue or **b** silver staining. The red box indicates distinct protein bands that were chosen for further analysis. Prestain marker PageRuler was used for size estimation

The snail mucus from the mantle collar and foot of *L. fulica* and *H. distincta* was separately collected and analyzed the protein profiles. The results showed common protein bands with some distinct bands (Fig. 1a). In addition, a gel with silver staining for *L. fulica* mucus was used to increase the sensitivity for detection of the mantle-specific bands, from which the band of about 30 kDa was selected (Fig. 1b). It should be noted that the reproducibility of this protein pattern might be varied from each sample collection, seasonal and location so the major protein bands which was more consistently observed were selected for further analysis.

### Identification of selected proteins by LC-MS/MS

The mucus from foot facilitates snail movement while the mantle mucus moistens the snail body, healing wounds and infections. Thus, for the purpose of medical and cosmetic applications, three distinct mantle-specific protein bands (30 kDa and 60 kDa from *L. fulica* and 56 kDa from *H. distincta*), shown in Fig. 1, were subjected to further analysis by LC-MS/MS. The results, matched with different known proteins (Additional file 3: Table S1) suggested that each band might be a mixture of proteins. Interestingly, the 30 and 60 kDa proteins were matched with achacin, the antimicrobial peptide in *L. fulica*, while the 56 and 60 kDa protein band from *H. distincta* and *L. fulica*, respectively, matched with different proteins including a sarcoplasmic calcium-binding protein (Scb).

### Biological activity of snail mucus

#### Antibacterial activity

**Fig. 2:**
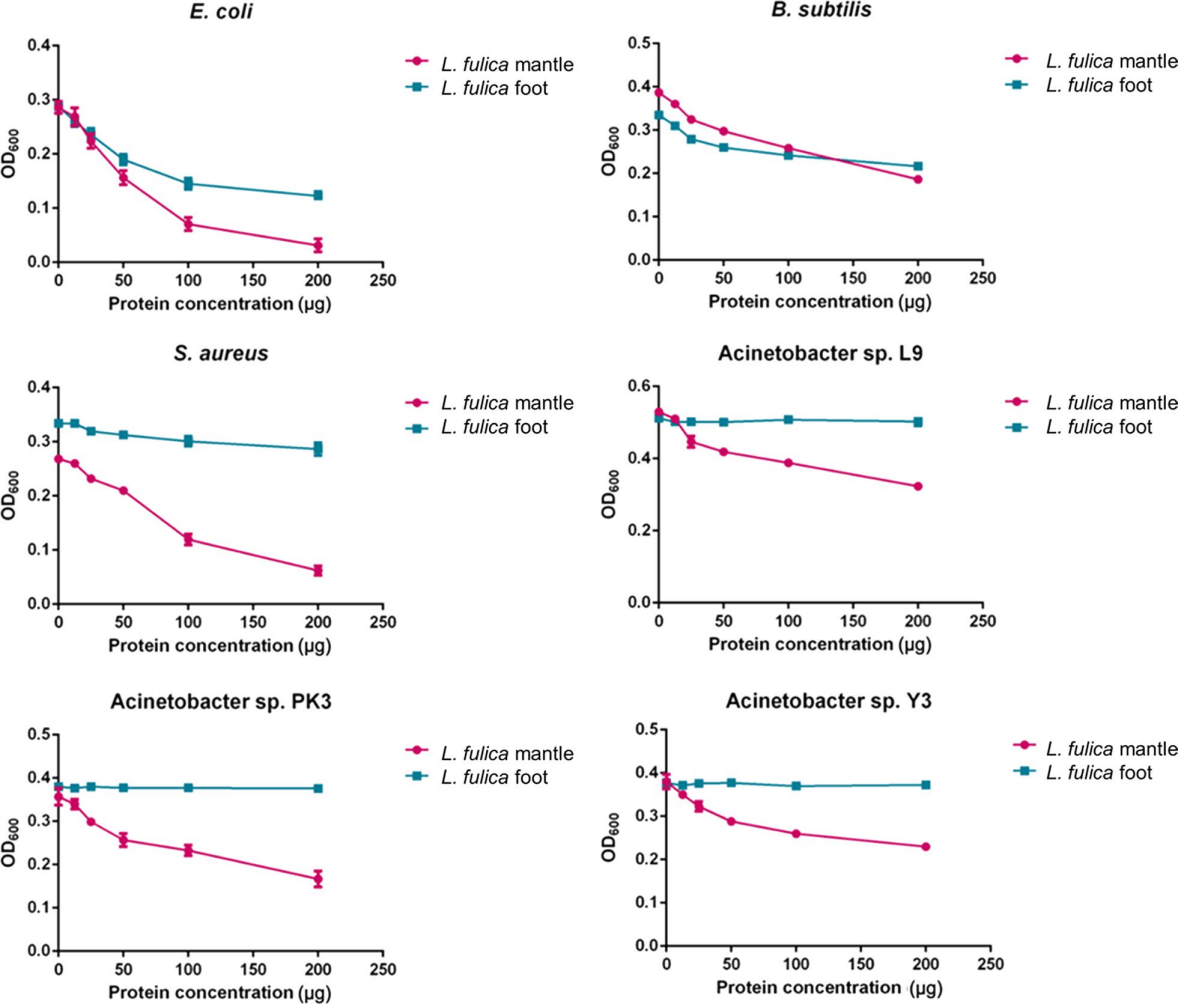
Antibacterial activity of *L. fulica* mucus. The different protein concentrations (12.5, 25, 50, 100, and 200 µg/100 µl PBS) were incubated with *E. coli*, *B. subtilis*, *S. aureus*, and *Acinetobacter* sp. L9, PK3, and Y3 overnight and then the OD_600_ was determined. Data are shown as the mean ± standard deviation, derived from three independent replicates

**Fig. 3:**
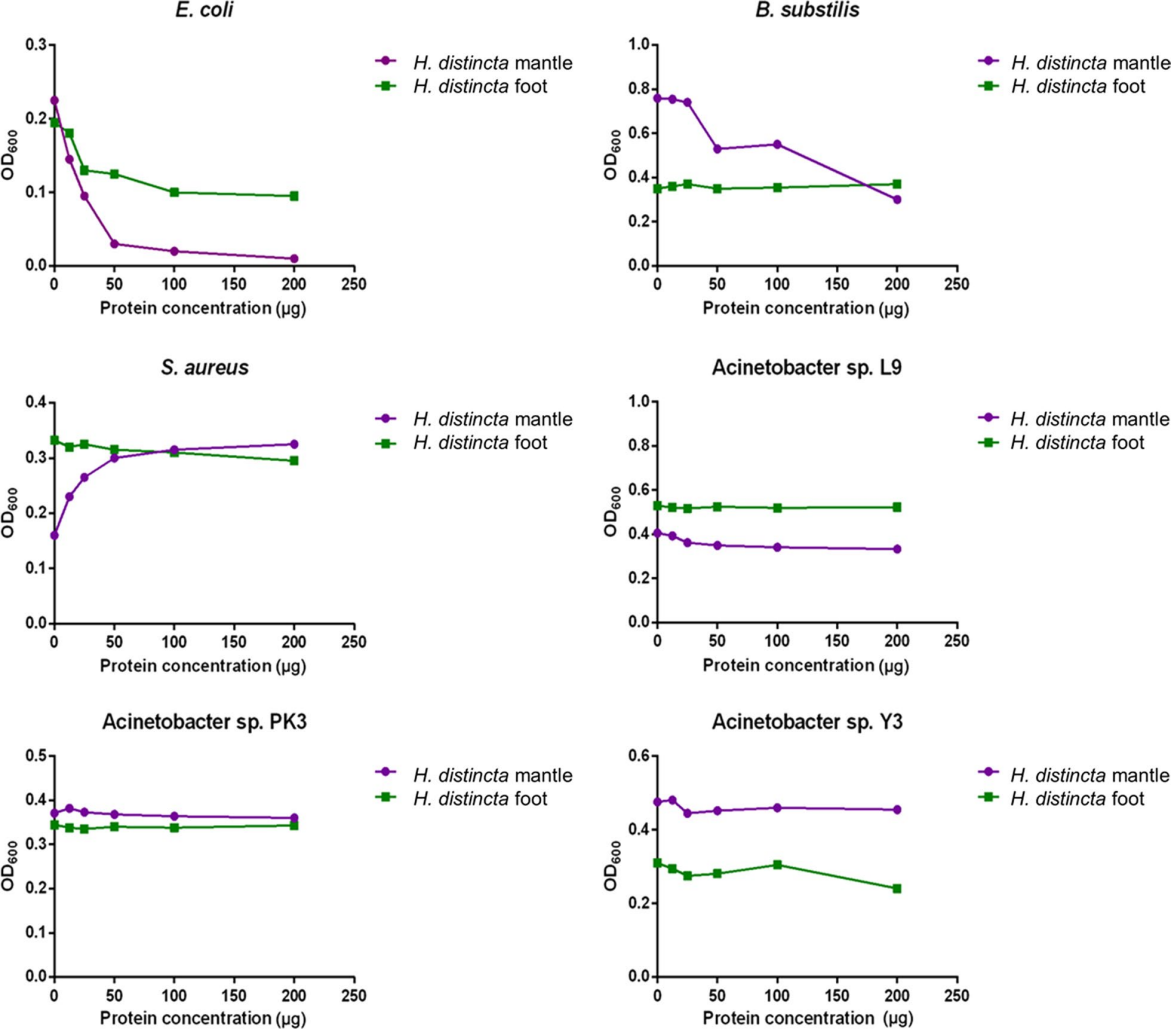
Antibacterial activity of *H. distincta* mucus. The different protein concentrations (12.5, 25, 50, 100, and 200 µg/100 µl PBS) were incubated with *E. coli*, *B. subtilis*, *S. aureus*, and *Acinetobacter* sp. L9, PK3, and Y3 overnight and then the OD_600_ was determined. Data are shown as the mean ± standard deviation, derived from three independent replicates

The antibacterial activity was tested against Gram-positive (*S. aureus* and *B. subtilis*) and Gram-negative (*E. coli* and *Acinetobacter* spp.) bacteria using a liquid broth inhibition assay. The mucus from the mantle of *L. fulica* exhibited antibacterial activity against all four tested bacterial strains while the mucus from the foot showed a lower activity against *E. coli* and *B. subtilis* only (Fig. 2). The mucus from the mantle of *H. distincta* could slightly inhibit *E. coli* and *B. subtilis* while the mucus from the foot has no antibacterial activity at the tested concentrations (Fig. 3). Overall, the results clearly demonstrated the higher antibacterial activity of the mucus from the mantle than that from the foot in both snail species.

#### Anti-tyrosinase activity

The *L. fulica* mucus had a significantly higher tyrosinase inhibition activity (32.3% and 33.4%) than the *H. distincta* mucus (14.7% and 16.5%) for both the mantle and foot mucus, respectively but the anti-tyrosinase activity of the mantle and foot mucus was not significantly different within a species for both snail species.

#### Antioxidant activity

The mucus from both snails showed an antioxidant activity, with a lower EC_50_ found in the mucus of *L. fulica*. The mantle mucus from *H. distincta* had a significantly higher EC_50_ (μg) of 129.9 ± 15.9 (less active) than that of the mantle 94.5 ± 4.3. For *L. fulica*, the EC_50_ (μg) between the foot (25.5 ± 2.9) and mantle (27.8 ± 5.2) were not significantly different.

## Discussion

It has previously been shown that different types of secretory glands discharge slime on to the surface of the mantle collar and foot of *Helix aspersa* [15]. As the slime performs various functions in the life of the snails, it is likely that snail slime compositions would vary according to the functional demands [22]. In this study, we found distinct protein patterns between the foot and mantle mucus within the same species and between the two different species of *L. fulica* and *H. distincta*.

For the purpose of medical and cosmetic uses, the mantle-specific protein bands were selected for further analysis by LC-MS/MS. Interestingly, one of the proteins identified from the selected distinct bands that was only observed in the *L. fulica* mantle mucus matched to achacin, an antibacterial protein [23–25]. This might reflect the strong antibacterial activity of *L. fulica* mantle mucus against all four tested bacteria, while the mucus from the foot that lacked this protein band could inhibit only the growth of *E. coli* and *S. aureus*. Previous studies have reported that the mucus from *L. fulica* and *H. aspersa* exhibited antibacterial activity against various strains of bacteria and fungi [26–30]. Moreover, several antimicrobial peptides (AMPs) from *L. fulica* mucus and *H. aspersa* have been investigated [25, 31–34]. Recently, the crude protein extracted from six snail species also showed antimicrobial activity against some bacteria and fungi [12].

Achacin is an antibacterial glycoprotein from the body surface mucus of *L. fulica*, which has been shown to exhibit a bactericidal effect [35]. In addition, the cDNA of achacin precursor was successfully cloned from the tissue of the snail collar [36]. Our study demonstrated that the mantle mucus of *L. fulica* may contain a higher relative activity of achacin than the foot mucus (which may contain none). The two bands of 60 and 30 kDa, identified as achacin suggest that the protein might function as a multi-subunit protein [15] or be in different states of glycosylation, etc. In this study, we also report for the first time the antimicrobial activity of the snail mucus from *H. distincta* but further investigations are needed to identify the antimicrobial molecule(s).

Related to the function of the mantle, the LC-MS/MS analysis also identified a mantle-specific protein band as a Scp, which was also not detected in the foot mucus. The mantle is muscular and forms the outer wall of the snail's body and secretes a mucus that contains a calcium-binding protein, which probably participates in the formation of calcium carbonate crystals to repair and maintain the shell of snail [37].

It has been shown that snail mucus from *H. aspersa* could inhibit the tyrosinase activity and melanin production on cell lines [11, 14]. In other snails, the extracts from different parts showed an antioxidant activity and the presence of many bioactive compounds [38–41]. In this study, we showed that the snail mucus from both *L. fulica* and *H. distincta* also exhibited an anti-tyrosinase as well as antioxidant activity. The biological activities of snail mucus indicate the beneficial effect on therapeutic and cosmetic applications.

In summary, the mucus secreted from the mantle and foot of *L. fulica* and *H. distincta* exhibits somewhat different protein compositions and biological assays clearly demonstrated that the mantle mucus exhibited a higher antibacterial activity than the foot mucus in both snail species. Various compounds in snail mucus need to be further characterized and clarified for their functions in the animal and the proper use in various applications.

## Limitations

Other components in the snail secretion besides proteins, were not analyzed in this study and they are likely to contribute also to the different biological activity of the snail secretion between foot and mantle.

## Supplementary Information

**Additional file 1:** **Figure S1**. Dorsal and ventral images of *Lissachatina fulica* and *Hemiplecta distincta*. The mantel collar and foot of the snails were indicated.

**Additional file 2:** **Figure S2**. Analysis of the main proteins in total snail mucus from *L. fulica* and *H. distincta* by SDS-PAGE.

**Additional file 3:** **Table S1**. List of proteins that matched the selected protein bands in snail mucus by LC-MS/MS analysis.

## Data Availability

All raw data are available from the corresponding author upon request.

## Abbreviations

SDS-PAGE: Sodium dodecyl sulphate-polyacrylamide gel electrophoresis
LC-MS/MS: Liquid chromatography/tandem mass spectroscopy
U: Unit
OD: Optical density
EC: Effective concentration
DPPH: 2,2′-diphenyl-1-picrylhydrazyl
L-DOPA: L-3,4-dihydroxyphenylalanine
