# Supplementary material for: Snail mucus from the mantle and foot of two land snails, Lissachatina fulica and Hemiplecta distincta, exhibits different protein profile and biological activity
Source: BMC Res Notes. 2021 Apr 15;14:138. doi: 10.1186/s13104-021-05557-0 (PMC8050916; doi:10.1186/s13104-021-05557-0)
Supplement: Supplementary file 1 — Additional file 1: Figure S1. Dorsal and ventral images of Lissachatina fulica and Hemiplecta distincta. The mantel collar and foot of the snails were indicated. [file 13104_2021_5557_MOESM1_ESM.docx]

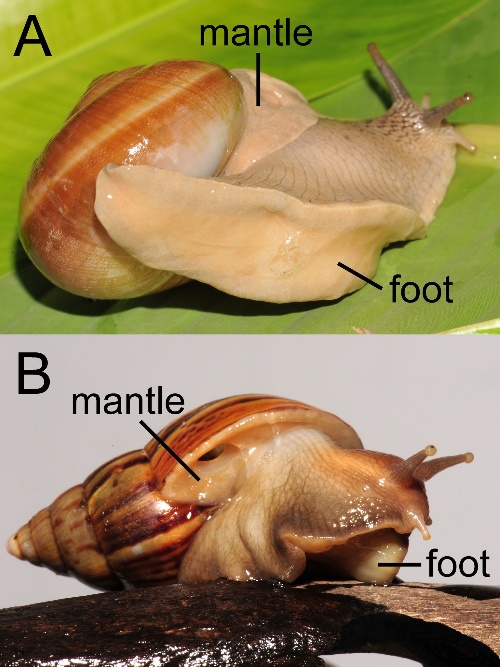


Additional file 1: Figure S1. Dorsal and ventral images of *Lissachatina fulica* and *Hemiplecta distincta*. The mantel collar and foot of the snails were indicated.
