# Supplementary material for: Snail mucus from the mantle and foot of two land snails, Lissachatina fulica and Hemiplecta distincta, exhibits different protein profile and biological activity
Source: BMC Res Notes. 2021 Apr 15;14:138. doi: 10.1186/s13104-021-05557-0 (PMC8050916; doi:10.1186/s13104-021-05557-0)
Supplement: Supplementary file 2 — Additional file 2: Figure S2. Analysis of the main proteins in total snail mucus from L. fulica and H. distincta by SDS-PAGE. [file 13104_2021_5557_MOESM2_ESM.docx]

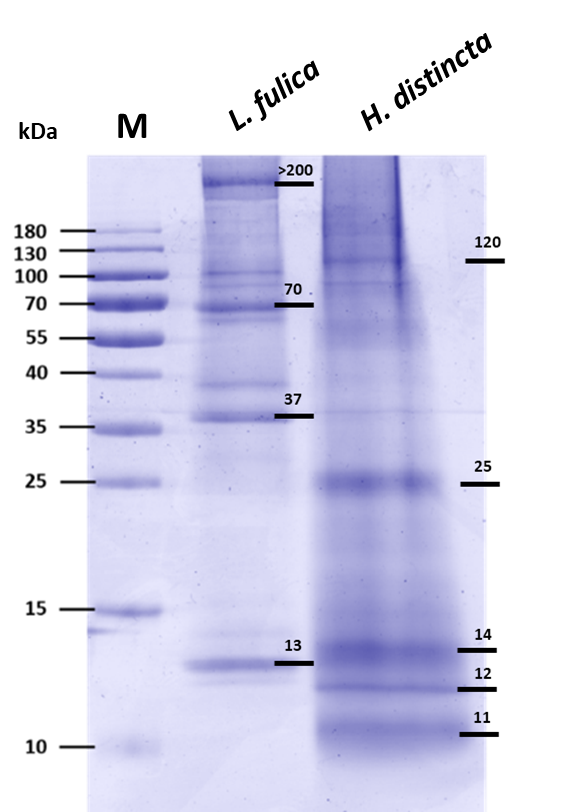


Additional file 2: Figure S2. Analysis of the main proteins in total snail mucus from *L. fulica* and *H. distincta* by SDS-PAGE. M; Prestain marker PageRuler. Mucus (25 µg/lane) was resolved by SDS-PAGE and then stained with Coomassie Brilliant Blue. Black lines indicate the major protein bands.
