# Supplementary material for: Snail mucus from the mantle and foot of two land snails, Lissachatina fulica and Hemiplecta distincta, exhibits different protein profile and biological activity
Source: BMC Res Notes. 2021 Apr 15;14:138. doi: 10.1186/s13104-021-05557-0 (PMC8050916; doi:10.1186/s13104-021-05557-0)
Supplement: Supplementary file 3 — Additional file 3: Table S1. List of proteins that matched the selected protein bands in snail mucus by LC-MS/MS analysis. [file 13104_2021_5557_MOESM3_ESM.docx]

Additional file 3: Table S1. List of proteins that matched the selected protein bands in snail mucus by LC-MS/MS analysis

| Size | Peptide seqence(s) | % identity | Accession | Description |
| --- | --- | --- | --- | --- |
| 56 kDa *H. distinca* | KLNSFSNYKV  VATVSLPR  KIATVIDPKS | 100  100  100 | YP_009406934  P86909.1  XP_012281726.1 | NADH dehydrogenase subunit 5 (mitochondrion) [*Typhlatya iliffei*]  Sarcoplasmic calcium-binding protein [*Chionoecetes opilio*]  uncharacterized protein C12orf31 homolog [*Orussus abietinus*] |
| 60 kDa  *L. fulica* | KFQENLNSKG  KSSTSHALTLNRK  KEFTEGFGKPGRT  KGELIPGSAPGANRV  RSETVNEAFNAVKF  KANQANLAGYYLKK  KLYQLTVDEALDKV  KGQTVELFEYSNRI  KSTSPFNQMYDWKS  KLTGFDGEVLTIPQANKL  KEELLSQLSQAYGIERS  RSVDVAVVGAGPSGTYSAYKL  KKLTGFDGEVLTIPQANKL  RLFTTHLPNVPDLNLESGGMRY  RKVILAIPQSALIHLDWKPLRS  VATVSLPR  KIKVLIAQKH  KLKVILGGGRL  KIQVIIKAKT  KIKVILGGGRK  KLQVLIKAKL | 100  100  100  100  100  100  100  100  100  100  100  100  100  100  100  100  100  100  100  100  100 | P35903.1  P86909.1  KXJ25145.1  ALS30424.1  XP_003738186.1  XP_018013387.1  XP_021961805.1 | Achacin; Flags: Precursor [*Lissachatina fulica*]  Sarcoplasmic calcium-binding protein [*Chionoecetes opilio*]  Proto-oncogene tyrosine-protein kinase receptor Ret [*Exaiptasia pallida*]  membrane-bound alkaline phosphatase isoform 2 [*Spodoptera frugiperda*]  nudC domain-containing protein 1 [*Galendromus occidentalis]*  alkaline phosphatase-like [*Hyalella azteca*]  uncharacterized protein LOC110857524 [*Folsomia candida*] |
| 30 kDa  *L. fulica* | RSETVNEAFNAVKF  KANQANLAGYYLKK  KLYQLTVDEALDKV  KGQTVELFEYSNRI  RLFTTHLPNVPDLNLESGGMRY  ILELESEKR  LAAEMPPK  LTVLEHLELVNALK  MFCIRLTK  ISSPELDER  EPLILRSGATVR  IWTECLNIR  HVFALLTRK  VVIVSIIGK  KISSNICQLR  MDTFGWSIEITAHDR  EQTINEIEK  SSSTPSPTSGWKR  HPCILKYISSWYR  IYYFDMMGRAEMTR  VASYDVSAIVMLNNVQEENK  ILNEVPTRPAELVKQQMTK  YKMMLFDFTNAQSVDDWR  LSSFASWPYTSKDR  NENLAALFGPPHPARLLPLGK  NNVGYTPLHAAVIK  QLLHTWGPAQRFLR  TVMIEFYDR  AQGNTLPCFHIPESR  RETTLWEEGDHAMGGIRPR  MHWNSANDSAPK  MIQYTGDGSK  NLLPMVEMIVKDHTR  LAPVSLDDAWK  NSLMMGR  VIVPPGEPMIVDGDGKR  FVSGANSKPELSERDR  ARPVLSGGSLVTGSYKWQIFSLIFLFIILQ | 100  100  100  100  100  100  100  100  100  100  100  100  100  100  100  100  100  100  100  100  100  100  100  100  100  100  100  100  100  100  100  100  100  100  100  100  100  100  100 | P35903.1 | Achacin; Flags: Precursor [*Lissachatina fulica*] |
